# Low Prevalence of Vitamin D Insufficiency among Nepalese Infants Despite High Prevalence of Vitamin D Insufficiency among Their Mothers

**DOI:** 10.3390/nu8120825

**Published:** 2016-12-21

**Authors:** Johanne Haugen, Manjeswori Ulak, Ram K. Chandyo, Sigrun Henjum, Andrew L. Thorne-Lyman, Per Magne Ueland, Øivind Midtun, Prakash S. Shrestha, Tor A. Strand

**Affiliations:** 1 Innlandet Hospital Trust, Lillehammer 2609, Norway; tors@me.com; 2 Centre for International Health, University of Bergen, Bergen 5007, Norway; 3 Department of Child Health, Institute of Medicine, Tribhuvan University, Kathmandu 8212, Nepal; manjeswori@gmail.com (M.U.); ram.chandyo@cih.uib.no (R.K.C.); prakashsunder@hotmail.com (P.S.S.); 4 Department of Nursing and Health Promotion, Oslo and Akershus University College of Applied Sciences, Oslo 0130, Norway; sigrun.henjum@hioa.no; 5 Johns Hopkins Center for Human Nutrition, Johns Hopkins Bloomberg School of Public Health, Baltimore, MD 21205, USA; athorne1@jhu.edu; 6 WorldFish, P.O. Box 500 GPO, Penang 10670, Malaysia; 7 Departments of Nutrition, Harvard T.H. Chan School of Public Health, Boston, MA 02115, USA; 8 Department of Clinical Science, University of Bergen, Bergen 5007, Norway; per.ueland@ikb.uib.no (P.M.U.); oivind.midttun@bevital.no (Ø.M.); 9 Bevital AS, Bergen 5021, Norway; 10 Department of Sports Science, Inland Norway University of Applied Sciences, Lillehammer 2604, Norway

**Keywords:** 25(OH)D, vitamin D status, season, lactating mothers, infants, Nepal

## Abstract

Background: Describing vitamin D status and its predictors in various populations is important in order to target public health measures. Objectives: To describe the status and predictors of vitamin D status in healthy Nepalese mothers and infants. Methods: 500 randomly selected Nepalese mother and infant pairs were included in a cross-sectional study. Plasma 25(OH)D concentrations were measured by LC-MS/MS and multiple linear regression analyses were used to identify predictors of vitamin D status. Results: Among the infants, the prevalence of vitamin D insufficiency (25(OH)D <50 nmol/L) and deficiency (<30 nmol/L) were 3.6% and 0.6%, respectively, in contrast to 59.8% and 14.0% among their mothers. Infant 25(OH)D concentrations were negatively associated with infant age and positively associated with maternal vitamin D status and body mass index (BMI), explaining 22% of the variability in 25(OH)D concentration. Global solar radiation, maternal age and BMI predicted maternal 25(OH)D concentration, explaining 9.7% of its variability. Conclusion: Age and maternal vitamin D status are the main predictors of vitamin D status in infants in Bhaktapur, Nepal, who have adequate vitamin D status despite poor vitamin D status in their mothers.

## 1. Introduction

Several studies have reported a high prevalence of vitamin D insufficiency and deficiency worldwide. Cultural practices may impact vitamin D status in otherwise healthy populations, best illustrated in cultures where women cover their bodies and heads and/or spend a lot of time indoors [1,2,3,4,5]. There are also reports of poor vitamin D status among otherwise healthy populations in Nepal [6,7,8], despite over 300 sunny days a year [9], and no cultural avoidance of the sun. The increasing awareness of possible “extra-skeletal effects” of vitamin D on outcomes such as development of malignant, cardiovascular, autoimmune, metabolic and infectious diseases [10], and pregnancy outcomes and child growth [11], has resulted in an ongoing debate about optimal levels of vitamin D and an increased effort to identify predictors and implications of poor vitamin D status.

Infants’ risk of developing vitamin D deficiency is often explained by limited sun-exposure and low vitamin D in breast milk [12,13,14,15,16]. Recent reports suggest that vitamin D content in breast milk is highly dependent on maternal vitamin D status [13,17], which in turn is dependent on synthesis during skin exposure to ultraviolet B radiation [18], and to a lesser extent also influenced by the inclusion of sources of vitamin D in the diet [19]. We have previously reported the micronutrient status of 500 infants in Bhaktapur where the prevalence of vitamin D insufficiency (cut-off < 50 nmol/L) was found to be as low as 3.6% [20]. Identification of predictors of 25(OH)D concentration in infants is important to prevent vitamin D insufficiency and deficiency. In this analysis we describe the status and predictors of vitamin D status in both the mother and infants included in the study, and explore the relationship between maternal and infant vitamin D status.

## 2. Materials and Methods

### 2.1. Ethical Clearance

Ethical clearances and approval of consent procedure were obtained from the ethics board of the Institute of Medicine in Tribhuvan University, Nepal (Ethical approval code 2064-10-17). Informed written consent was obtained from the participating mothers. The study was conducted according to the guidelines provided in the Declaration of Helsinki [21].

### 2.2. Study Design and Participants

**Figure 1:**
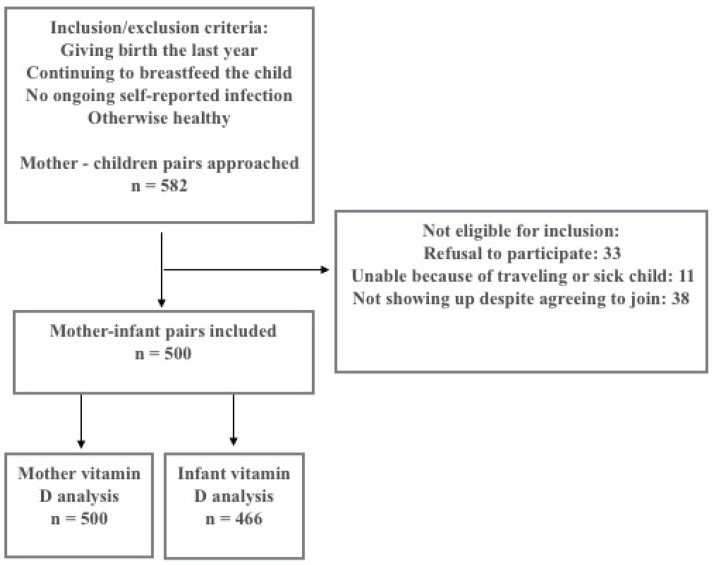
Study flow chart.

A cross-sectional survey on micronutrient intake and status was conducted in 500 healthy lactating women (17–44 years old) and their breastfed infants (1–12 months) during 2008–2009 [22,23]. The mothers and infants were recruited from Bhaktapur municipality (27°42′ N), Nepal, which is a peri-urban area with a total population of approximately 75,000, made up of mostly farmers, semi-skilled or unskilled laborers and daily wage earners. The study was designed to detect a prevalence of 25% for any given micronutrient deficiency. With an absolute precision of 4% i.e., a 95% confidence interval (CI) ranging from 21% to 29%, we estimated that 450 included mothers would be adequate. With an expected incomplete sampling of 10%, the final sample size was set to 500. We used a two-stage cluster sampling procedure whereby 66 neighborhoods (“toles”) were randomly selected as the primary sampling unit from a total of 160. We listed all women living in these toles and randomly selected 582 women. The study flow chart is presented in Figure 1.

In total, 582 mothers were approached and 82 of them were not included due to refusal to participate (*n* = 33), because of other reasons such as travelling or sick child (*n* = 11), or not showing up despite agreeing to join the study (*n* = 38). Inclusion criteria for the mothers were giving birth during the last 12 months, still lactating (either full or partly), willing to provide household information and consent to participate. Exclusive breastfeeding was defined as no other foods or drinks than breast milk, although treatment for medical purposes was accepted. Exclusion criteria were self-reported ongoing infection in the mother or in the infant, which was clinically verified by physical examination.

### 2.3. Data Collection

The mothers and their children came to Siddhi Memorial Hospital for physical examination, anthropometric measurements and blood draws between January 2008 and February 2009. Numbers of mother and infant pairs included by month were distributed throughout the year as follows: January (44), February (30), March (35), April (31), May (44), June (45), July (62), August (49), September (30), October (25), November (48) and December (57). Type and timing for complementary foods given to the infants were registered. Trained fieldworkers performed three 24 h recalls for the subjects on different weekdays and daily average intake of energy and different nutrients was estimated. Procedures of the dietary recalls and estimation of macro- and micronutrient intake with results are thoroughly described elsewhere [24]. Anthropometric measurements were obtained using UNICEF weighing scale and a calibrated locally produced height board (Salter; SECA, Hamburg, Germany). Stunting, wasting and underweight were defined using the World Health Organization (WHO) criteria. The criteria for stunting was height for age <−2 SD of the WHO Child Growth Standards median, the criteria for wasting was weight for height <−2 SD and the criteria for underweight was weight for age <−2 SD [25]. BMI for the mothers was calculated as weight/height^2^ (kg/m^2^) and BMI < 18.5 was considered underweight, 18.5–25.0 as normal weight and >25.0 as overweight.

### 2.4. 25(OH)D Analysis and Other Laboratory Tests

Venous non-fasting blood samples drawn from a cubital vein from 500 mother and infant pairs were collected into micronutrient-free, heparinized polypropylene tubes (Sarstedt). 25(OH)D was analyzed from 466 of the infants only due to insufficient plasma in some of the samples. The blood samples were taken from mother and infant within the same week. Hemoglobin was measured immediately by using the HemoCue 201 system (HemoCue, Vedbæk, Denmark). After centrifuging the samples at 760 g for 10 min at room temperature, plasma was transferred into micronutrient-free, heparinized polypropylene vials (Eppendorf, Hinz, Germany) and stored at −70 °C before transport on dry ice to Norway and then stored at −80 °C at Innlandet Hospital Trust in Lillehammer for three years prior to analysis [22]. It is expected that 25(OH)D samples would be stable under such conditions [26]. The mother and child samples were kept in the same environment and equally treated in all stages of the study. C-reactive protein (CRP) was measured as part of the description of the population and as a marker for ongoing infection, and was analyzed by turdibimeric immunoassay (Tina-Quant, Roche, Berlin, Germany) on a Hitachi (Tokyo) at the Laboratory for Clinical Biochemistry at Haukeland University Hospital, Bergen. Vitamin analyses were performed by BEVITAL (www.bevital.no), Bergen. Liquid chromatography-tandem mass spectrometry (LC-MS/MS), which is the current preferred analysis method [27], was used for 25(OH)D analysis. The measurement of total plasma 25(OH)D included the two isoforms 25(OH)D_2_ and 25(OH)D_3_ [28]. The method did not separate C3-epimers of 25(OH)D_2_ and 25(OH)D_3_ from the non-epimeric forms.

### 2.5. Outcome Variables and Definition of Vitamin D Status

The primary outcome of this report was vitamin D status measured as total plasma 25(OH)D among the mothers and their infants. Defining sufficient and deficient levels of vitamin D is controversial. We used the recommendations from the review report “Dietary Reference Intakes for Vitamin D and Calcium” from 2011 published by the U.S. Institute of Medicine to describe vitamin D status [29]. Skeletal outcomes are used to define the cut-off of ≥50 nmol/L, which is referred to as sufficient to maintain bone health for >97% of the population. In the same report, concentrations between 30 and 50 nmol/L are reported to be associated with maximum calcium uptake in the age group 19–50 years of age, and levels below 30 nmol/L are reported to be associated with increased risk of vitamin D deficiency rickets in infants. These considerations are the basis for using the cut-offs <50 nmol/L (referred to as suboptimal/insufficient) and <30 nmol/L (referred to as deficient) to describe vitamin D status in both the mothers and the infants in our paper. Recognizing the ongoing debate over thresholds used to define vitamin D status, we also present the proportion <75 nmol/L as this cut-off often is used to reflect marginally vitamin D insufficiency [30].

### 2.6. Meteorological Characteristics of the Study Area and Estimation for UVB Effect

There are four definite climatic seasons in this area. Pre-monsoon season spans from March to May with a hot and humid climate; monsoon from June to September with >80% of the annual rainfall; post-monsoon from October to November, and winter from December to February. The winter is characterized with a dry cold climate with minimum temperatures below 0 °C and daily maximum temperatures of approximately 12 °C. During summer temperatures may reach 35 °C [31]. Since skin-synthesis of vitamin D_3_ in response to UVB-radiation is the most important source of vitamin D, sun-exposure has a huge impact on vitamin D status. Exact sun-exposure and hours spent in the sunshine was not recorded in the study, so to estimate the variation in UVB effect, we used a quantitative measure for sunshine, mean daily solar radiation due to month, measured in MJ/m^2^/day. We did not directly measure global solar radiation, but estimated monthly global solar radiation using existing models based on available parameters such as sunshine duration, maximum and minimum temperature, relative humidity, rainfall and geographical location [9]. 25(OH)D has a three week half-life and there is therefore a lag in detecting the exact relationship between sun-exposure and 25(OH)D production. In order to express the association between global solar radiation and 25(OH)D we used the mean of the last three months global solar radiation, including the month the blood sample was taken.

### 2.7. Statistics

Data were analyzed using Stata version 14. Means with standard deviations or medians with interquartile range (IQR) were used for descriptive statistics. Pearson’s correlation coefficient was used for estimating the correlation between maternal and infant 25(OH)D concentrations. Multiple linear regression was used to identify predictors of vitamin D status. We adjusted the confidence intervals and *p*-values for the cluster design of the study. We selected the variables manually (purposeful selection), as described by Hosmer and Lemeshow [32]. For infant 25(OH)D concentration, the following variables were included in this process: infant age (in months), sex (male/female), weight-for-age and weight-for-height z-scores, birth weight, exclusively breastfeeding, maternal body mass index, maternal total plasma 25(OH)D, occupational status (no work or agricultural/other work) mean global solar radiation and CRP. For maternal 25(OH)D concentration; maternal age (years), maternal BMI, CRP, blood pressure, parity, occupational status and mean global solar radiation were included as candidate variables in the multiple regression analyses.

## 3. Results

### 3.1. Baseline Infant and Mother Characteristics

Baseline characteristics of the infants are shown in Table 1.

IQR = inter quartile range. The median (IQR) age was 7 (4–9) months and 55.4% of the children were boys. The prevalence of stunting, wasting and underweight were 9.7%, 1.9% and 5.5%, respectively. Of the infants included, 72 (14.8%) were currently exclusively breastfed with a mean (± SD) age of 3.4 ± 1.4 months. Baseline characteristics of the mothers are shown in Table 2. Mean (± SD) age was 25.8 ± 4.2 years and only 10 (2%) were reportedly taking vitamin or mineral supplements, though the vitamin D content of those supplements was not determined in this study. Mother characteristics are presented in Table 2. Mean (± SD) energy-intake was estimated from the three 24 h dietary recalls to be 2089.3 kcal ± 428.8/day. Intake of dietary vitamin D_2_ and D_3_ was not possible due to incomplete food composition-tables.

### 3.2. Vitamin D Status

The overall mean (±SD) 25(OH)D concentration in the infants was 82.0 ± 21.4 nmol/L and 449 (96.4%) had sufficient 25(OH)D levels (≥50 nmol/L). Only 17 (3.6%) of the infants had total plasma 25(OH)D (<50 nmol/L), only 3 (0.6%) had <30 nmol/L and 191 (41.0%) <75 nmol/L (Table 3).

Of the infants, 96 (20.6%) had detectable 25(OH)D_2_, and only 2 of these were exclusively breastfed. Of the mothers, only 12 (2.4%) had detectable 25(OH)D_2_. 25(OH)D_2_ accounted for 5.7% and 0.8% of the total 25(OH)D concentration in infants and mothers, respectively. The mean (± SD) total plasma 25(OH)D in the group of currently exclusively breastfed infants was 87.1 ± 19.0 nmol/L and none of these infants had vitamin D levels <50 nmol/L. The overall mean (± SD) total maternal plasma 25(OH)D was 47.4 ± 16.4 nmol/L and 201 (40.2%) had sufficient concentrations. The number with plasma 25(OH)D <50 nmol/L was 299 (59.8%) (Table 3), 70 (14.0%) had 25(OH)D <30 nmol/L and 476 (95.2%) had levels <75 nmol/L.

**Figure 2:**
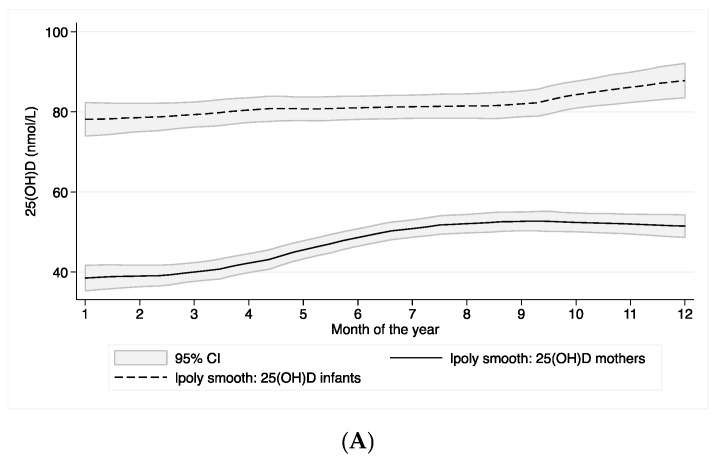

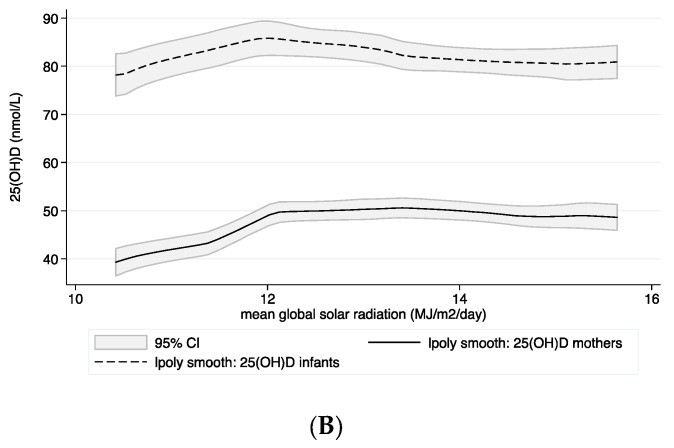
Infants and mothers 25(OH)D, and variability with month of the year (**A**) and global solar radiation (**B**). (**A**) 25(OH)D concentration by month 1–12 of the year (January–December) for infants (upper line) and mothers (lower line); (**B**) 25(OH)D concentration by global solar radiation for infants (upper line) and mothers (lower line).

The concentrations varied substantially by season for the mothers but not for the infants. Figure 2 shows the concentration of 25(OH)D according to months of the year (A) and solar radiation (B).

### 3.3. Predictors for Plasma 25(OH)D Concentrations in Infants and Mothers

The variables predicting plasma 25(OH)D concentrations in infants are shown in Table 4.

The infants’ vitamin D status was positively associated with maternal vitamin D status and maternal BMI, and inversely associated with age of the infant. Exclusive breastfeeding did not modify the association between maternal 25(OH)D and infant 25(OH)D. The variables explained 22% of the variability in the infant’s vitamin D status.

Variables predicting maternal vitamin D status are shown in Table 5.

Maternal vitamin D status was associated with mean global solar radiation, maternal age and BMI. These variables explained 9.7% of the variability of the mothers’ vitamin D status. Adjusting for cluster design did not change the precision of our estimates or any of the *p*-values substantially. Maternal vitamin D status was associated with season and highest during the monsoon and post-monsoon seasons when solar radiation was at its highest.

## 4. Discussion

In this study, maternal vitamin D status and infant age were important predictors for the infants’ vitamin D status, while solar radiation and mothers’ age were the most important predictors for maternal vitamin D status. Surprisingly, the prevalence of vitamin D insufficiency in the infants was very low compared to the mothers, despite the positive association between the maternal and infant 25(OH)D concentrations. This low prevalence is surprising when compared with other populations, including studies from neighboring India [1,2,3,4,5].

UVB-radiation of the skin converts 7-dehydro-cholesterol to pre-vitamin D_3_, which then undergoes thermal isomerization to form vitamin D_3_. Dietary sources of vitamin D_3_ and vitamin D_2_ are limited to foods such as oily fish and some vegetables such as mushrooms, but both vitamin D_2_ and vitamin D_3_ are available in supplements and enriched foods. Vitamin D_3_, and vitamin D_2_ undergoes hydroxylation reactions in the liver to form 25(OH)D_3_ and 25(OH)D_2_ (calcidiol) and then the kidneys to form the active vitamin D metabolite, 1,25(OH)_2_D (calcitriol). Breast milk is suggested to meet the nutritional needs of infants younger than six months, although traditionally portrayed as a poor source of vitamin D [33,34]. However, recent studies suggest that breast milk is adequate in vitamin D when the mother has sufficient circulating concentrations of vitamin D_2_ and/or vitamin D_3_ [13,17,35]. The vitamin D_3_ content of the breast milk is also reported to increase many times compared to 25(OH)D_3_ in the mothers’ serum after sun-exposure [36]. Because of the short half-life of vitamin D_3_, the breastfeeding mother needs daily intake or dermal synthesis of vitamin D in order for her breast milk to have adequate vitamin D for her infant. Maternal transfer of vitamin D_3_ to the infant with subsequently lower production of maternal 25(OH)D could be one explanation for the difference in maternal and infant vitamin D status. The negative association between vitamin D status and infant age in our study, with a likely higher breastfeeding frequency among younger versus older children, and our finding of vitamin D sufficiency in the exclusively breastfed group, could also indicate that breast milk is a good source of vitamin D in this population.

Differences in vitamin D status between infants and mothers could also be due to supplementation of vitamin D_2_ and/or vitamin D_3_ through the diet. Vitamin D supplementation is not currently recommended for infants in Nepal and until now the use of other supplements and infant formulas has been uncommon [37]. All the infants in our study were breastfed, but most of them received additional foods, either homemade complementary foods or processed complementary foods such as “Lito” and “Cerelac”. The content of 25(OH)D in these foods were not recorded, but it is plausible that some of this food was supplemented with vitamin D. However, in contrast to previously reported results from India, United Arab Emirates, Pakistan and Greece [1,2,3,5,38], all exclusively breastfed infants (*n* = 70) in this study were also vitamin D sufficient, indicating a substantial contribution to vitamin D status from other than supplementary sources. Due to insufficient food tables we could not estimate dietary vitamin D_2_ and vitamin D_3_ intake for the women. Only 10 women reported regular intake of vitamin or mineral supplements, but the vitamin D content of these supplements could not be determined. 25(OH)D_2_ was not detectable in any of the women taking supplements, indicating no supply of vitamin D_2_ from these supplements. The detectable 25(OH)D_2_ in 12 women indicates a possible vegetable source (or other supplemental source) of vitamin D_2_ for these women.

Some studies report an inverse relationship between markers of inflammation and vitamin D status [39,40,41]. Acknowledging this relationship, we included CRP as a candidate predictor variable, although this was a healthy population without clinical infection or inflammation. We did not find any correlation between CRP and 25(OH)D in this study, and the median CRP concentration was higher in the infants than in the mothers. Decreasing levels of 25(OH)D during pregnancy has also been reported [42] and could be a plausible explanation for low maternal 25(OH)D after delivery, but the existing evidence is conflicting [43,44].

Vitamin D_3_ from dermal synthesis due to UVB-radiation is an important source of vitamin D, and a frequently reported cause of vitamin D insufficiency is avoidance of the sun due to socioeconomic and/or cultural factors. In Nepal there is no cultural avoidance of the sun and rather a tradition of sunbathing. In some areas of Nepal there is a tradition of outdoor breastfeeding, with subsequent infant sun-exposure, and these habits have previously been thought to be associated with vitamin D status in Nepalese children [6]. Outdoor breastfeeding habits could be a possible explanation for the good vitamin D status in the infants. Additionally, the tradition in the Bhaktapur area of sunbathing the babies while massaging them in oil would also definitely contribute to the skin-synthesis of vitamin D_3_ in the infants. Sun-exposure was not precisely investigated, but differences in sun-exposure are a possible reason for vitamin D sufficiency among infants and insufficiency among the mothers. The capacity of vitamin D_3_ synthesis has also been reported to be to some extent dependent of the age of the skin, which could contribute to differences [45].

The positive associations between maternal BMI and 25(OH)D concentration is difficult to interpret. In a recent meta-analysis there was a weak, but significant negative correlation between BMI and 25(OH)D concentration among adults [46]. The authors concluded, however, that more research is needed to verify the relationship in populations living in developing regions. Higher maternal intake of energy-rich food containing vitamin D could have been a possible explanation for higher 25(OH)D concentrations, but energy-intake was not associated with maternal, nor infant 25(OH)D concentrations in this study.

The strengths of this study are the high response rate from a large representative sample of lactating women and their breastfed infants, as well as the number of specimens obtained. There are several limitations, however. Sun-index data and related factors including time spent in the sun, covering with clothes and skin pigmentation, were not collected. Although exact sun-exposure-indexes are difficult to obtain, this could have added useful information regarding vitamin D sources. The IOM report recommend that vitamin D intake should cover the daily requirements of vitamin D, and discourages to rely on sunlight exposure to produce vitamin D in the skin in any population, even in southern climates with abundant sunshine [29,47]. The vitamin D contents of foods and supplements could not be determined due to insufficient food tables. According to the recommendation of covering vitamin D requirements from dietary sources, we therefore cannot draw any conclusions regarding the necessity of vitamin D supplementation from these results. Finally, the different assays used for measuring plasma- or serum-25(OH)D have different strengths and weaknesses. As most commercial LC-MS/MS-assays, the assay used in this study, did not estimate C3-epimers, and an overestimation in the 25(OH)D concentration due to inactive C3-epimers can therefore not be excluded [48].

A general challenge when estimating vitamin D status is the lack of consensus about definitions of sufficiency and deficiency, especially in children. The cut-offs used in this paper are based on the U.S. Institute of Medicine (IOM) consensus report from 2011 [29], which, in agreement with the Pediatric Endocrine Society [49], targeted a serum value for 25(OH)D of at least 50 nmol/L as sufficient for a healthy bone metabolism in nearly all children and adults. Other experts report values <50 nmol/L as deficiency and <75 to 80 nmol/L as “insufficient”, even for children [30]. The cut-off of 50 nmol/L is based upon the associations between 25(OH)D concentration and skeletal outcomes. Aside from the increased risk of rickets observed at very low levels of vitamin D and/or calcium status among infants, we have little evidence to guide us on what a “sufficient” level of vitamin D in infants is. To describe vitamin D deficiency for the infants in our study, we also considered using the cut-off of <37.5 nmol/L, which was recommended in an article from the Pediatric Endocrine Society [49]. The prevalence of “vitamin D deficient” infants in our study was, however, exactly similar for this cut-off as for the cut-off <30 nmol/L. We therefore decided to consistently use the IOM consensus report tresholds, and to keep the cut-off similar for both mothers and infants.

## 5. Conclusions

In conclusion, in this study in a healthy population of lactating mothers and their infants, maternal vitamin D status and infant age were important predictors of infant vitamin D status, and season and maternal age were predictors of maternal vitamin D status. Despite a significant correlation between maternal and infant 25(OH)D concentrations and a high prevalence of vitamin D insufficiency among the mothers, the prevalence of vitamin D insufficiency was low among the infants. Maternal transfer of vitamin D_3_ through breast milk and differences in sun-exposure may be possible explanations for the difference in 25(OH)D concentrations. More research is also needed to elucidate the consequences of postpartum maternal deficiency.

## Figures and Tables

**Table 1:** Infant characteristics in a nutritional survey in mother and infant pairs in Bhaktapur, Nepal.

Characteristics	Value	*n*
Age in months, median (IQR)	7 (4–9)	487
Boys, *n* (%)	277 (55.4)	500
Number of siblings, mean ± SD	1.8 ± 0.9	498
Months of known excl. breastfeeding, median (IQR)	3 (2–5)	486
Weight kg, mean ± SD	7.5 ± 1.3	479
Length/height cm, mean ± SD	66.3 ± 4.9	479
Wasted (<−2 SD weight for length), *n* (%)	9 (1.9)	476
Underweight (<−2 SD weight for age), *n* (%)	26 (5.5)	477
Stunted (<−2 SD length for age), *n* (%)	46 (9.7)	475
Birth weight recall/card grams, mean ± SD	2891.5 ± 491.8	438
C-reactive protein in mg/L, mean ± SD	4.3 ± 10.9	449
Currently exclusively breastfed, *n* (%)	72 (14.8)	486

**Table 2:** Mother/(parent) characteristics in a nutritional survey in mother and infant pairs in Bhaktapur, Nepal.

Characteristics	Value	*n*
Age years, mean ± SD	25.8 ± 4.2	499
Current regular vitamin or mineral supplement use, *n* (%)	10 (2.0)	499
Folic acid supplements during pregnancy, *n* (%)	115 (23.0)	500
Iron supplement in pregnancy, *n* (%)	449 (89.8)	500
Calcium supplement in pregnancy, *n* (%)	402 (80.4)	500
Other medication in pregnancy, *n* (%)	54 (10.8)	500
Weight kg, mean ± SD	50.9 ± 7.6	499
Height cm, mean ± SD	150.5 ± 5.4	499
BMI, mean ± SD	22.5 ± 3.1	499
Diastolic blood pressure, mean ± SD	75.0 ± 8.4	499
Systolic blood pressure, mean ± SD	111.0 ± 10.4	499
Mother’s energy-intake/day kcal, mean ± SD	2089.3 ± 428.8	466
C-reactive protein mg/L, mean ± SD	1.7 ± 6.5	500
Mother working outside home, *n* (%)	122 (26.3)	464
Father working outside home, *n* (%)	431 (93.5)	461
Mother’s education 10. grade or more, *n* (%)	220 (47.4)	464
Father’s education 10. grade or more, *n* (%)	298 (64.1)	465

**Table 3:** Vitamin D status of infants and their mothers in a nutritional survey in Bhaktapur, Nepal.

Vitamin D Status	Infants		Mothers	
	Value	*n*	Value	*n*
Total 25(OH)D in nmol/L, mean ± SD	82.0 ± 21.4	466	47.4 ± 16.4	500
Detectable 25(OH)D_2_, *n* (%)	96 (20.6)	466	12 (2.4)	500
25(OH)D_2_ in nmol/L, mean ± SD	4.7 ± 12.9	466	0.4 ± 2.7	500
Detectable 25(OH)D_3_, *n* (%)	466 (100)	466	500 (100)	500
25(OH)D_3_ in nmol/L, mean ± SD	77.3 ± 22.0	466	47.0 ± 15.5	500
Total 25(OH)D in nmol/L, excl. breastfed, mean ± SD	87.1 ± 19.0	70	53.0 ± 20.2	72
25(OH)D_3_ in nmol/L, excl. breastfed, mean ± SD	86.6 ± 19.5	70	51.8 ± 16.6	72
Total 25(OH)D in nmol/L ≥50 nmol/L, *n* (%)	449 (96.4)	466	201 (40.2)	500
Total 25(OH) in nmol/L <50 nmol/L, *n* (%)	17 (3.6)	466	299 (59.8)	500
Total 25(OH)D in nmol/L <30 nmol/L, *n* (%)	3 (0.6)	466	70 (14.0)	500
Total 25(OH)D in nmol/L <75 nmol/L, *n* (%)	191 (41.0)	466	476 (95.2)	500

**Table 4:** Predictors for plasma 25(OH)D (nmol/L) concentrations in a random sample of Nepalese infants.

Covariate	Coeff. Crude	95% CI	*p*	Coeff. Adj.	95% CI	*p*	β *	R^2^
Age of child (months)	−2.0	−2.6, −1.3	<0.001	−1.5	−2.1, −0.9	<0.001	−0.2	0.22
Mothers 25(OH)D (nmol/L)	0.5	0.4, 0.6	<0.001	0.5	0.4, 0.6	<0.001	0.4	
Mothers BMI (kg/m^2^)	0.9	0.3, 1.5	0.004	0.6	0.02, 1.2	0.041	0.08	

*N* = 464. The other variables tested in crude models: gender, z-scores, birth weight, CRP, duration exclusively breastfeeding, maternal average energy-intake, global solar radiation, and mothers occupational status were not significantly associated with the children’s 25(OH)D concentration. * standardized regression coefficients

**Table 5:** Predictors for plasma 25(OH)D (nmol/L) concentrations in a random sample of Nepalese lactating women.

Covariate	Coeff Crude	95% CI	*p*	Coeff. Adj.	95% CI	*p*	β *	R^2^
Mothers BMI (kg/m^2^)	0.3	−0.1, 0.6	0.109	0.6	0.2, 1.0	0.002	0.1	0.097
Global solar radiation (MJ/m^2^/d)	2.6	1.8, 3.4	<0.001	2.6	1.8, 3.4	<0.001	0.3	
Mothers age (years)	−0.6	−0.9, −0.2	0.002	−0.7	−1.1, −0.4	<0.001	−0.2	

*N* = 499. The other variables tested in the crude models: blood pressure, CRP, maternal average energy-intake, parity status and occupational status were not significantly associated. * standardized regression coefficients.
